# Strategies for Efficient Gene Editing in Protoplasts of Solanum tuberosum Theme: Determining gRNA Efficiency Design by Utilizing Protoplast (Research)

**DOI:** 10.3389/fgeed.2021.795644

**Published:** 2022-01-20

**Authors:** Frida Meijer Carlsen, Ida Elisabeth Johansen, Zhang Yang, Ying Liu, Ida Nøhr Westberg, Nam Phuong Kieu, Bodil Jørgensen, Marit Lenman, Erik Andreasson, Kåre Lehmann Nielsen, Andreas Blennow, Bent Larsen Petersen

**Affiliations:** ^1^ Department of Plant and Environmental Sciences, Faculty of Science, The University of Copenhagen, Copenhagen, Denmark; ^2^ Kartoffel Mel Centralen Amba, Brande, Denmark; ^3^ Department of Cellular and Molecular Medicine, Faculty of Health Sciences, University of Copenhagen, Copenhagen, Denmark; ^4^ Department of Plant Breeding, Swedish University of Agricultural Sciences, Alnarp, Sweden; ^5^ Department of Plant Protection Biology, Swedish University of Agricultural Sciences, Alnarp, Sweden; ^6^ Bioscience, Aalborg University, Aalborg, Denmark

**Keywords:** ribonucleoprotein/gRNA design, gene editing, protoplast, CRISPR/Cas, complex genome

## Abstract

Potato (*Solanum tuberosum*) is a highly diverse tetraploid crop. Elite cultivars are extremely heterozygous with a high prevalence of small length polymorphisms (indels) and single nucleotide polymorphisms (SNPs) within and between cultivars, which must be considered in CRISPR/Cas gene editing strategies and designs to obtain successful gene editing. In the present study, in-depth sequencing of the glucan water dikinase (GWD)1 and the downy mildew resistant 6 (DMR6-1) genes in the potato cultivars Saturna and Wotan, respectively, revealed both indels and a 1.3–2.8 higher SNP prevalence when compared to the heterozygous diploid RH genome sequence as expected for a tetraploid compared to a diploid. This complicates guide RNA (gRNA) and diagnostic PCR designs. High editing efficiencies at the cell pool (protoplast) level are pivotal for achieving full allelic knock-out in tetraploids and for reducing the downstream cumbersome and delicate ex-plant regeneration. Here, CRISPR/Cas ribonucleoprotein particles (RNP) were delivered transiently to protoplasts by polyethylene glycol (PEG) mediated transformation. For each of GWD1 and DMR6-1, 6–10 gRNAs were designed to target regions comprising the 5′ and the 3′ end of the two genes. Similar to other studies including several organisms, editing efficiency of the individual RNPs/gRNAs varied significantly, and some generated specific indel patterns. While RNPs targeting the 5′ end of GWD1 yielded significantly higher editing when compared to targeting the 3′ end, editing efficiencies in the 5′ and 3′ end of DMR6-1 appeared to be somewhat similar. Simultaneous targeting of either the 5′ or the 3′ end with two RNPs (multiplexing) yielded a clear positive synergistic effect on the total editing when targeting the 3′ end of the GWD1 gene only. Multiplexing of the two genes, residing on different chromosomes, yielded no or slightly negative effects on the individual RNP/gRNA editing efficiencies when compared to editing efficiencies obtained in the single RNP/gRNA transformations. These initial findings may instigate larger studies needed for facilitating and optimizing precision breeding in plants.

## Introduction

High ploidy and complex genomes are hallmarks of many crops and plant species. Furthermore, outcrossing, selfing, and more general non-hybrid breeding crops, are often highly heterozygous (Labroo et al., 2021; Bamberg et al., 2021; Farrell et al., 2014). Successful gene editing in such species, as well as the following regeneration/propagation of the edited ex-plant, pose several challenges, including *1)* attainment of full allele-specific sequence information within the target region of the gene of interest; *2)* implementation of an efficient and robust high throughput method for scoring editing at the protoplast (cell pool) and ex-plant level; *3)* selection and efficiency scoring of guide RNAs (gRNAs), *4)* selection of target regions within genes/genomes that confer the highest editing efficiency *in vivo*; *5)* devising high efficiency multigene (multiplexing) targeting methodologies in order to reduce the number of edited plant regeneration rounds, which in most cases includes tissue culturing and *6)* establishment of ex-plant regeneration schemes for crop plants. We have previously contributed to developing schemes and solutions for *i-iii)* (Petersen et al., 2019; Johansen et al., 2019; Bennett et al., 2020) and partly to *6)* (Andersson et al. (2018); Nicolia et al. (2015)). Here we address *4)* and *5)*, making a first investigation into the effect of choice of target region for gRNAs in a gene of interest in relation to single gRNA/Cas9 ribonucleoprotein (RNP) and multiplexed RNP editing efficiencies.

Most potato cultivars are tetraploid and have a complex genome with many genes, often consisting of a high number of small exons distributed over large regions (The Potato Genome Sequencing Consortium et al., 2011; Pham et al., 2020). A huge number of both single nucleotide polymorphisms (SNPs) and length polymorphisms (indels) are general hallmarks of tetraploid potato cultivars (The Potato Genome Sequencing Consortium et al., 2011; Pham et al., 2020). Previously, in-depth sequence analysis of the start of the Granular Bound Starch Synthase (GBSS) gene in the tetraploid cultivars Desiree and Wotan showed a significantly higher prevalence of single nucleotide polymorphisms (SNPs) and included indels (Johansen et al., 2019) when compared to the heterozygous diploid *S. tuberosum* group RH89-039-16 (The Potato Genome Sequencing Consortium et al., 2011). To ensure complete editing of all allelic variants in a gene locus full allele-specific sequence analysis of the target region in question is therefore required prior to gRNA, as well as for diagnostic PCR designs.

While CRISPR derived off-target events have attained much focus in the public and scientific debate, unintentional small genetic changes (somaclonal variation) associated with cell and tissue culture, where hormones are used to develop the entire plant (ex-plant) from a single protoplast cell, have been shown to be significantly higher than mutations from CRISPR derived off-target events (Tang et al., 2018; Li et al., 2019). Repeated rounds of tissue culturing should thus ideally be avoided, and it is therefore desirable to generate all required mutations by multiplexing gene editing for the desired trait changes in a single transformation event. To ensure generation of a high number of ex-plants with multiple full allelic mutated target genes, each individual RNP should confer a high editing efficiency. For example, if two RNPs each confer 10% full allelic editing at the protoplast cell pool level, one out of 100 plants will have all alleles (8 in total) in both target genes edited. Including a third gene, only one in 1,000 plants will have full editing of all loci.

Many factors may influence the editing efficiency of a given RNP, including GC content and primary and secondary structure of the gRNA (Liu et al., 2020; Bortesi et al., 2016). Additionally, heterochromatin parts of chromosomes have been shown to be edited less efficiently than euchromatic or actively transcribed genes (Chechik et al., 2020). Also, DNA and histone and methylation have been shown to influence the chromatin state through gene imprinting (Chechik et al., 2020; Schubeler, 2015) and thus regulate gene expression. Many active promoter regions are nucleosome-free regions (Xu et al., 2009; Jin et al., 2009), which allows easier access for the transcriptional machinery to assemble and initiate transcription. More generally, it was found that an open chromatin structure is more frequently occurring in the 5′ end of genes (Li et al., 2007) compared to the middle and 3’ end, which has a more tightly packed chromatin status (Schubeler, 2015). Probably due to this and other factors influencing RNP efficiency (Grajcarek et al., 2019), CRISPR-Cas editing activity has been shown to vary significantly as a result of both gRNA structure and target region (Sansbury et al., 2019).

In potato, agrobacterium delivered dual gRNAs, targeted at single genes and thus enabling PCR-based deletion screening, was shown to confer efficient gene editing in the potato cultivars Desiree and King Edward (Kieu et al., 2021). However, possible position effects were not included in the study, and a more systematic comparison of gRNAs targeting the same or separate genes has to our knowledge, not been carried out.

The glucan water dikinase (GWD) 1 is a key regulatory enzyme in starch metabolism (Mahlow et al., 2016). GWD1 phosphorylates amylopectin rendering it more susceptible to degrading hydrolytic enzymes (Hebelstrup et al., 2015). Overexpression of GWD1 in rice has resulted in elevated yields and has been suggested as an ideal biotechnological target for improving yield and quality in rice (Wang et al., 2021). The active site histidine and the hypothetic redox regulatory ‘CFATC’ sequence are encoded by exons 24 and 25 (Mikkelsen et al., 2005). The downy mildew resistant 6 (DMR6-1) gene encodes a 2-oxoglutarate (2OG) and Fe(II)-dependent oxygenase, which has a salicylic acid (SA) 5-hydroxylase activity and thus reduces the active SA pool. The DMR6-1 gene is classified as a susceptibility (S) gene, which is important for successful pathogen infection. Loss of function of DMR6-1 confers broad-spectrum disease resistance in tomato (Thomazella et al., 2021) and increased resistance to *Phytophthora infestans* in potato (Kieu et al., 2021). The catalytic domain and iron-binding residues H212, D214, and H269 of DMR6-1 are encoded by exon 3 and 4 (Van Damme et al., 2008).

In the present study, we performed allele-specific in-depth sequencing of two target areas comprising the 5′ end and the last third (here defined as 3′ end) of each of the GWD1 and the DMR6-1 genes in the potato cultivars Saturna and Wotan, respectively. 3–5 RNPs/gRNAs were targeted to each of the 5′ and the 3′ end of the two genes, and significantly higher editing was found when targeting the 5′ end as compared to the 3′ end of the GWD1 gene only. Also, when two RNPs/gRNAs were simultaneously applied (multiplexing), both targeting one of the regions in each of the genes, a positive synergistic effect on the total editing was only found when targeting the 3′ region of the GWD1 gene. Multiplexed targeting the 3’ region of both genes, residing on different chromosomes, did seemingly not confer synergistic effects.

## Material and Methods

### Plant Material

*In vitro* grown plantlets of Wotan and Saturna were obtained from Vitroform (Årslev, Denmark) and propagated in a Fitotron growth cabinet with 16/8 h, 24 °C/20 °C, 70% humidity, at 65 μE light intensity as described in Johansen et al., 2019 (Johansen et al., 2019).

### Gene Structure Including gRNA Target Regions of Glucan Water Dikinase *StGWD1*

*StGWD1* (Soltu.DM.05G009520.2) in the Phureja DM1-3 v.61 reference genome is located on chromosome five and comprises 33 exons (http://solanaceae.plantbiology.msu.edu/index.shtml) (chr05:9901255.9916669) (9902080.9902337, 9903053.9903094, 9903210.9903319, 9903416.9903494, 9903585.9903676, 9903758.9903911, 9904899.9905123, 9905242.9905583, 9905676.9905783, 9905896.9905994, 9906315.9906455, 9906583.9906671, 9907205.9907339, 9907551.9907713, 9907822.9907911, 9907986.9908171, 9909578.9909640, 9909715.9909777, 9909857.9909916, 9910010.9910162, 9910280.9910363, 9911005.9911181, 9911267.9911373, 9911740.9911953, 9912034.9912137, 9912482.9912614, 9912751.9912876, 9913879.9913989, 9914293.9914391, 9914746.9914919, 9915193.9915285, 9915518.9915633, 9915990.9916194). gRNAs were designed to match exon 1: 9902080.9902337 (5′ end); exon 24: 9911740. 9911953 and exon 25: 9912034.9912137 (3′ end). Genbank accession number for GWD1 (*XP_006357619.1*).

### Gene Structure of Downey Mildew Resistant (2-Oxoglutarate (2OG) and Fe(II)-Dependent Oxygenase Superfamily Protein) 6 (DMR6-1)

*StDMR6-1* (Soltu.DM.03G021450.3) in the Phureja DM1-3 v.61 reference genome is located on Chromosome three and comprises four exons (chr03:46,329605.46336003); (46335699.46335896, 46335367.46335614, 46330610.46330934, 46330195.46330437). gRNAs were designed for exon 1 and 2 (46335699.46335896, 46335367.46335614) (5′end) and exon 3 (46330610.46330934) (3′ end). Genbank accession number *DMR6-1* (XP_006347521.1).

### Genomic DNA Extraction

gDNA extractions were performed using GenElute Plant genomic DNA miniprep kit from Sigma (cat# G2N10) and quantified using UV spectroscopy (IMPLEN NP80) and stored at -80°C. Protoplasts were lyzed by snap freezing in liquid nitrogen followed by incubation at 96°C for 15 min and stored at-20°C.

### Allele Specific Genomic Sequences

Plant leaves from Saturna and Wotan were shipped to BGI (Shenzen, China) and sequenced to >80X coverage using DNBSeq and >30X coverage using PacBio long-read sequencing. Reads were mapped to DM1-3 v4.03 using CLC Genomics Workbench v20. Resolution of allele specific sequences was made by manual inspection of the target regions. Variants, including SNP and small indels, were called using the default settings.

### Diagnostic Indel Detection Amplicon Analysis (IDAA) PCR Primer and Guide RNA Design

The allele specific sequences, together with the variant calls across alleles, were used as the foundation for the design of diagnostic PCR and gRNA. Primer pairs for diagnostic PCR screening were generated using NCBI Primer-BLAST (https://www.ncbi.nlm.nih.gov/tools/primer-blast/). NCBI Primer-BLAST failed to generate suitable primer designs for the selected regions in DMR6-1, hence primers for the 5’ end of DMR6-1 were designed manually. Primer sequences and other specifications are summarized in Table 1.

**TABLE 1:** gRNAs and diagnostic IDAA primers for each of the four target regions. Scores and first selection of gRNAs were obtained by feeding ca 1 kb regions to the *in silico* prediction servers CHOPCHOP (http://chopchop.cbu.uib.no/), CRISPRater (https://crispr.cos.uniheidelberg.de/) and SSC (http://crispr.dfci.harvard.edu/SSC/).

	Diagnostic PCR(s)	gRNA
GWD—5′ exon 1	GWD Forward primer 1	gJ: TCA​GTG​GTA​AGT​ACA​GCA​TG
	5′ TTT​GTA​TTG​ACT​GAT​TTT​GTA​TTG​T 3′	gK: AGG​GAA​TAA​CTT​GCT​GTA​CC
	GWD Reverse primer 1	gL: GTT​TCG​AGG​TAA​CAG​GTT​AA
	FAM 5′ TAG​TTT​CTA​AGC​CCC​AAG​CA3′	gM: GTA​CAG​CAA​GTT​ATT​CCC​TA
GWD—3′ exon 24 + 25	GWD Forward primer 2:	gA: GGA​GAG​GAG​GAA​ATT​CCT​GA
	5′ TCA​GTC​CAG​TTG​AAG​CCG​TTG 3′	gB: TGT​TCG​AGC​TAG​AAA​TGG​GA
	GWD Reverse primer 2:	gC: GCT​GAC​CTC​CAA​GCA​AAG​GA
	FAM 5′ TCA​CGA​GTT​CAT​TCA​TCT​TTC​CCA 3′	gD: ATT​GGC​TGA​CCT​CCA​AGC​AA
		gE: TTT​CTG​TTC​GAG​CTA​GAA​AT
		gI: CAC​AAC​GAC​AAC​ATA​TCC​AA
DMR6—5′ exon 1	DMR6 Forward primer 1	g43: TTT​GAG​GGA​GAG​TAG​AGT​GG
	FAM 5′ CCA​TGG​AAA​CGA​AAG​TTA​TTT​C 3′	g44: GTG​GCC​TAT​CGG​ATT​CGG​GT
	DMR6 Reverse primer primer 1	
	5′ CAA​CCT​AAG​TCA​ATT​ATT​GGA​AC 3′	
DMR6—5′ exon 2	DMR6 Forward primer 2	g45: TGG​AGA​AAT​ATG​CTC​CTG​AA
	*5′ AGC​TGA​CCG​GCA​GCA​AAA​TTG*GGT​AGC​TGG​GGA​ATT​TTT​CA 3′	
	DMR6 Reverse primer 2	
	5′ GGT​TAC​CAT​GCA​TAA​CTA​TAC​ACA​C 3′	
	FAM primer	
	FAM 5′ AGC​TGA​CCG​GCA​GCA​AAA​TTG 3′	
DMR6—5′ exon 1 + 2	DMR6 Forward primer 1	g43: TTT​GAG​GGA​GAG​TAG​AGT​GG
	FAM 5′ CCA​TGG​AAA​CGA​AAG​TTA​TTT​C′3	g44: GTG​GCC​TAT​CGG​ATT​CGG​GT
	DMR6 Reverse primer 2	g45: TGG​AGA​AAT​ATG​CTC​CTG​AA
	5′ GGT​TAC​CAT​GCA​TAA​CTA​TAC​ACA​C 3′	
	DMR6 Reverse primer 4	
	FAM 5′ CGA​TGG​ATT​AGA​AGG​CCA​TTC 3′	
DMR6—3′ exon 3	DMR6 Forward primer primer 3	g46: GAA​GCC​ATA​GCA​GAG​AGC​CT
	5′ ATC​GTG​AGC​AGA​TAT​TGC​ACG 3′	g47: GAA​TTT​GGA​TCA​GTA​TGG​GC
	DMR6 Reverse primer 3	g48: ATC​ACC​AAG​ATT​AAT​GAC​AA
	FAM 5′ GGT​TTA​CCT​GCA​ATT​GAT​CAC 3′	

### Protoplast Isolation and Transformation

Protoplast isolation and transformation were essentially done as described in (Nicolia et al., 2015) using 10 min incubation in 25% w/v PEG4000 (Sigma) for transformation.

### Indel Detection Amplicon Analysis (IDAA)

Indel Detection Amplicon Analysis (IDAA) (Yang et al., 2015) was performed as described in (Johansen et al., 2019; Kemp et al., 2017) with FAM labelled primers as specified in Table 1. Amplifications of the DMR6-1 and GWD1 regions were done using CloneAmp HiFi PCR premix (Takara Bio cat#639298) in a 25 µL reaction with 0.25 µM primer and 1-2 µL protoplast suspension. PCR cycle parameters for the DMR6-1 5′ end region and both GWD1 regions were 5 min at 94°C followed by 40 cycles of 10 s at 98°C, 15 s at 60°C and 1 min at 72°C, followed by 5 min at 72°C. PCR cycle parameters for DMR6-1 3’ end region was 2 min at 98°C followed by 35 cycles of 10 s at 98 °C, 10 s at 55°C, 8 s at 72°C. Labeled PCR products were analyzed as outlined in (Yang et al., 2015). Editing efficiency was calculated from the peak areas in the IDAA chromatogram using the online software VIKING (https://viking-suite.com/).

### Ribonucleoprotein (RNP) Preparation

For each transformation, RNP was assembled by mixing 37.5 pmol modified TrueGuideTM (Invitrogen) with 37.5 pmol (2.5 µg) TrueCutTM Cas9 v.2 (Invitrogen) and incubating the mix for 12–16 h at 4°C. RNPs were then transferred to room temperature (appx 25 °C) and gently mixed with 100 µL 1.6 × 10^6^ protoplasts/mL followed by addition of 100 µL 25% w/v PEG solution, which was mixed by tapping the tube. The transformation was stopped after 10 min. For twin RNP combinations, 37.5 pmol of each RNP was mixed just before transformation.

## Results

In the present study, we selected two agriculturally relevant genes, the glucan water dikinase (GWD) 1 and the downy mildew resistant 6 (DMR6-1) gene in the commercial potato cultivars Saturna and Wotan, respectively, for assessing and devising schemes for scoring high efficiency gRNA targets that potentially generate full allelic editing in single protoplast cells.

### Characterization of the Target Regions in GWD1 and DMR6-1

**FIGURE 1:**
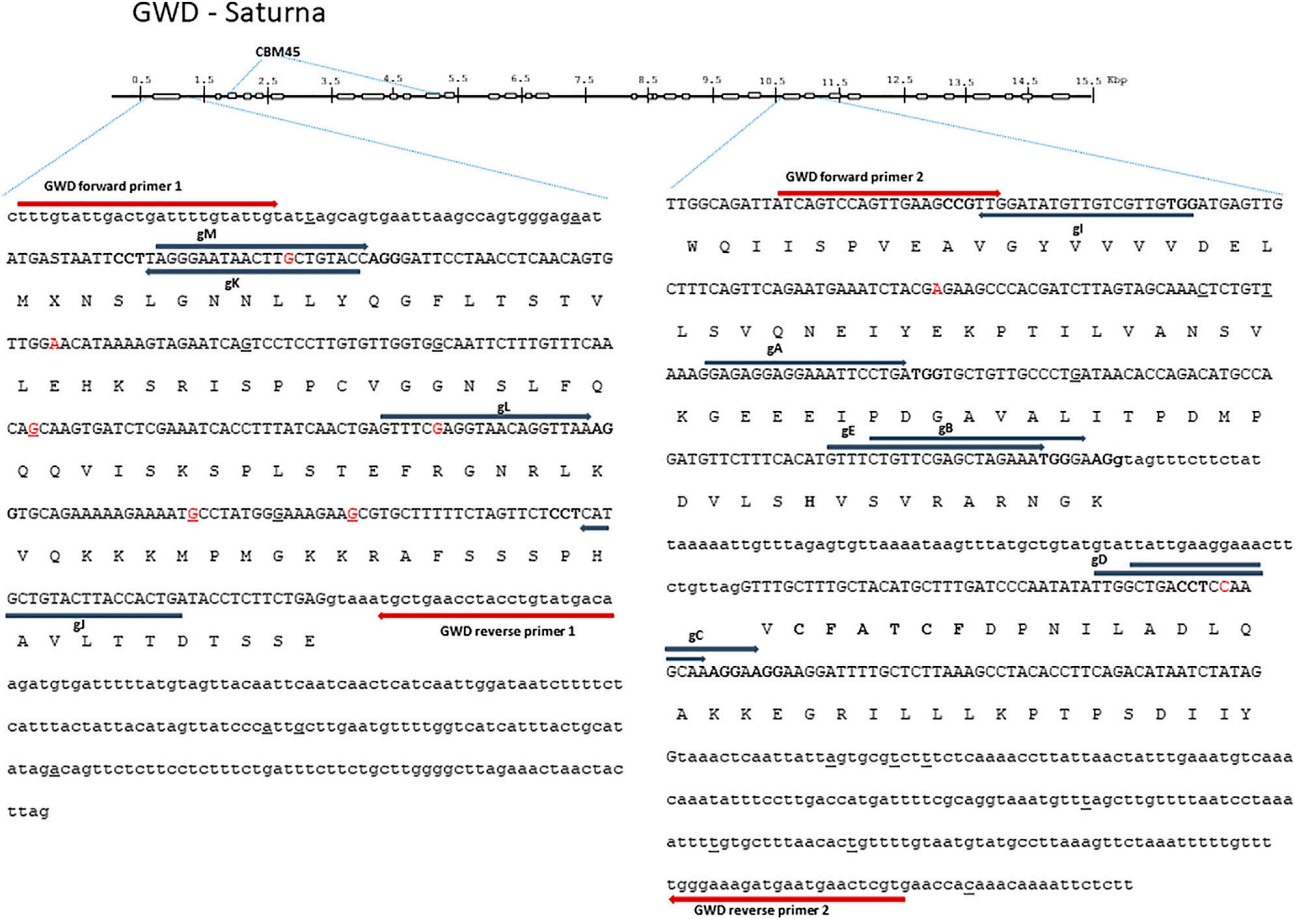
Glucan Water Dikinase (GWD) 1—structure and full allelic sequence of gRNA target regions. Overall gene structure with exons (boxes) and the area containing carbohydrate binding module (CBM) depicted above. Left: the nucleotide sequence of exon 1 and introns. Right: exon 24 and 25 including introns. Exons are depicted in capital letters with the amino acid sequence indicated. Small nucleotide polymorphisms (SNPs) from cultivars included in the SPUD database are marked with red, and SNPs found in Saturna are underlined. Grey arrows designate gRNAs (gA, gB, gC, gD, gE, gI, gJ, gK, gL, and gM) with PAM sites marked in bold. Red arrows designate diagnostic IDAA PCR primers. The “CFATC” region, containing cysteine’s hypothesized to be involved in inter or intra-disulfide bond formation and thus in putative redox-state modulation of GWD activity is marked with bold. The active site histidine residue is also marked with bold.

**FIGURE 2:**
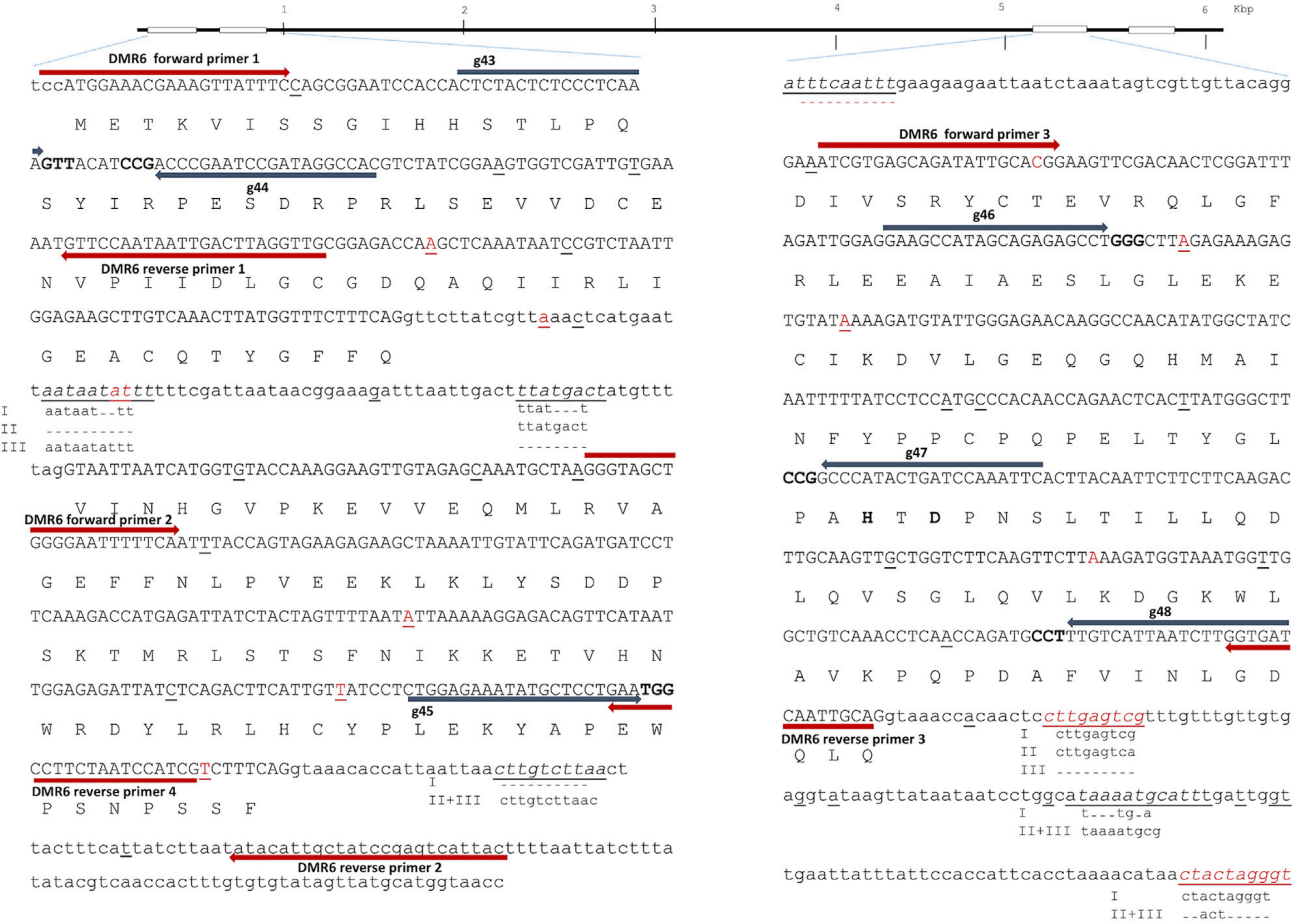
Downey Mildew Resistant (DMR) 6—structure and full allelic sequence of gRNA target regions. Overall gene structure with exons (boxes) is depicted above. Left: the nucleotide sequence of exon 1 and 2 with introns. Right: Exon 3 including introns. Exons are depicted in capital letters with the amino acid sequence indicated. Small nucleotide polymorphisms (SNPs) from cultivars included in the SPUD database are marked with red, and SNPs found in Wotan are underlined. Grey arrows designate gRNAs (g43, g44, g45, g46, g47, and g48) with PAM sites marked in bold. Red arrows designate diagnostic IDAA PCR primers. Two of the three catalytic residues histidine H212 and aspartic acid D214 marked with bold. The last catalytic residue H269 located in exon 4 is outside of the figure.

GWD1 and DMR6-1 are encoded by 33 and four exons and located on chromosome five and three in the Solanum tuberosum L. genome (http://solanaceae.plantbiology.msu.edu/), respectively. Start, i.e., exon 1 in GWD1 and exon 1 and 2 in DMR6-1, as well as last third, i.e. active site encoding exon 24 and 25 in GWD1 and exon 3 in DMR6-1, of the two genes were chosen as editing target regions and are here designated 5′ and 3′ end, respectively (Figures 1 and 2). The target region of the GWD1 gene includes the ‘CFATC’ region, containing cysteines hypothesized to be involved in inter or intra-di-sulfide bond formation and thus in putative redox-state modulation activity of GWD, and the residue histidine in the active site (Adegbaju et al., 2020; Ritte et al., 2006; Mikkelsen et al., 2005) (Figure 1). Catalytic site residues of DMR6-1 are histidine H212, and H269 as well as aspartic acid D214 (Zeilmaker et al., 2015) (Figure 2).

In-depth long read and Sanger sequencing were applied to map SNPs in all the alleles of GWD1 in Saturna (Figure 1) and DMR6-1 in Wotan (Figure 2). We found a 2.8 and 1.3 fold increased SNPs prevalence in the target exons of the two genes (Hamilton et al., 2011; Uitdewilligen et al., 2015), respectively, and also indels when compared to heterozygous diploid *S. tuberosum* group Tuberosum RH89-039-16, underscoring the high heterozygosity of elite potato cultivars. The SNPs and indels posed constraints on the placement of gRNAs and diagnostic PCR Indel Detection Amplicon Analysis (IDAA) primers (Figure 1).

### Diagnostic InDel Detection Amplicon Analysis (IDAA) PCR Designs

In order to meet the demand for fast and robust scoring of editing in high ploidy complex genomes and multiplex settings, we earlier implemented Indel Detection Amplicon Analysis (IDAA) for scoring editing in potato and tobacco (Petersen et al., 2019; Johansen et al., 2019; Bennett et al., 2020). Here, editing was scored by IDAA using FAM-primer labeled PCR products that were placed to avoid WT SNPs and indels (Figures 1 and 2).

### Ribonucleoprotein (RNP) Delivery and Initial gRNA Selection

gRNAs were designed by subjecting the four target regions to the *in silico* prediction server platforms CHOPCHOP (http://chopchop.cbu.uib.no/), CRISPRater (https://crispr.cos.uni-heidelberg.de/), SSC(http://crispr.dfci.harvard.edu/SSC/).

We used RNP for the various editing experiments delivered to protoplasts by polyethylene glycol (PEG) transformation. Equimolar gRNA and Cas9 RNP components were incubated ON at 4 °C, to enable RNP complex formation, and in case of experiments with combined RNPs, the two ON incubated RNP’s were mixed just before the transformation event, in order to reduce the theoretical risk of RNP re-complexing (see material and Methods).

### *In vivo* Efficacy of Individual and Combined RNPs/gRNAs

**FIGURE 3:**
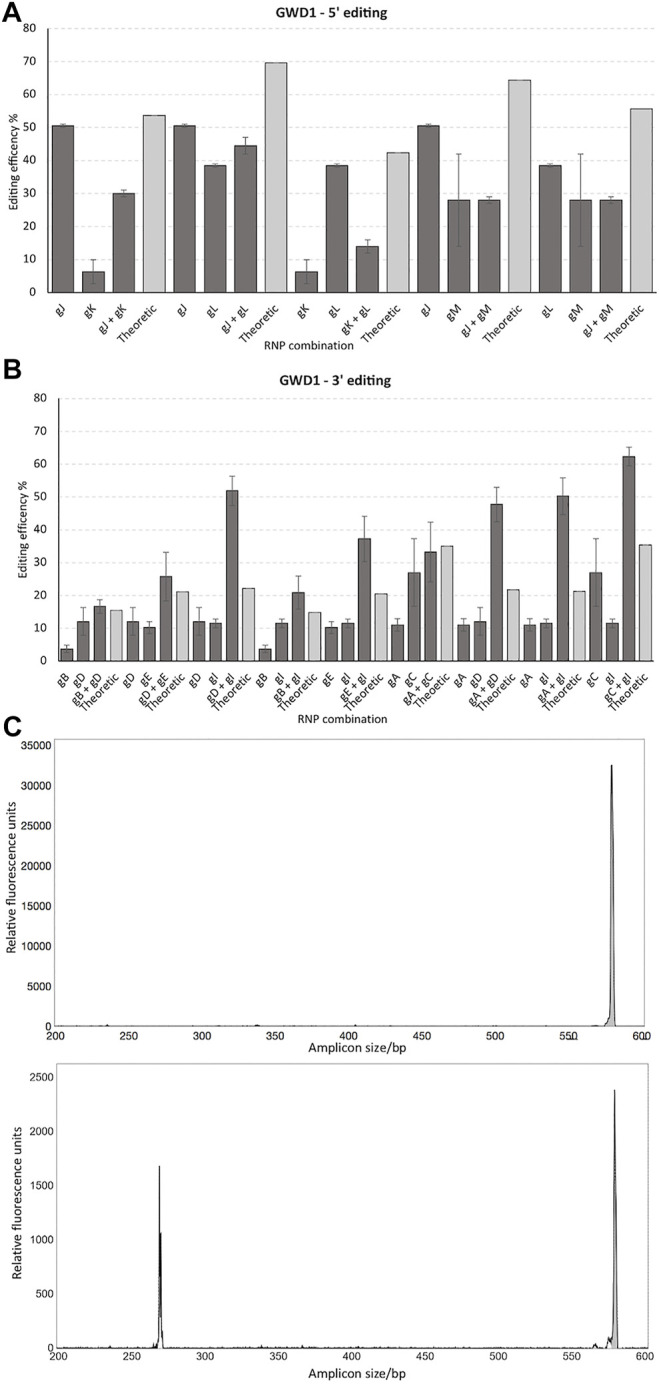
Targeting the 5′ versus the 3′ end of GWD1 and effect of multiplexing. 4 and 6 gRNAs were designed for the 5’ (gJ, gK, gL, and gM) **(A)** and 3’ (gA, gB, gC, gD, gE, and gI) **(B)** end ofGWD1, respectively. The gRNAs were tested individually and in combination. Theoretical designates the probability of editing, calculated as the multiplied probabilities of each gRNA not conferring editing, (1 – gRNA1 editing fraction) * (1 – gRNA2 editing fraction) subtracted from 1, i.e., 1 – [(1 – gRNA1 editing fraction) * (1 – gRNA2 editing fraction)] * 100 (%), where 1 designates all alleles in the cell pool. **(C)**: IDAA chromatogram of WT amplicon (upper panel, grey) and gD and gI combined editing (lower panel) including the unedited WT amplicon (grey) andminor editing from either gDor gI and where the lower sized amplicons include the combined editing of gD and gI andthus the gD and gI delineated deletion. The X-axis and Y-axis show the size of the amplicons in bp and relative fluorescence, respectively. IDAA primer pairs used for scoring gA, gB, gC, gD, gE & gI: GWD Forward primer 1 + GWDReverse primer 1 and for scoring gJ, gK, gL & gM: GWD forward primer 2 + GWD reverse primer 2 (see *Materials and Methods*).

Four gRNAs targeting the 5′ end of GWD1 showed from 6% to 52% editing of all alleles in the cell pool, as demonstrated by IDAA (Figure 3A). In this region, the combined total editing resulting from the use of two RNPs in the same transformation (multiplexing) did not exceed the editing of the best of the two RNPs when transformed individually (Figure 3A).

Editing efficiencies resulting from the application of twin RNP did not exceed the editing of the better of the two RNPs, and a somewhat negative synergism in the total editing (the sum of editing’s from both RNPs in the same transformation) appeared to result from multiplexing in this region (Figures 3A,B). This could suggest that Cas9 binding to one genomic site is interfering with binding of a second Cas9 at adjacent sites, possibly by steric hindrance.

Targeting the 3′ end of GWD1 as compared to targeting the 5′ end showed generally lower editing efficiency, ranging from 3–27% editing of alleles in the cell pool, as evidenced by IDAA. Combining two RNPs in this region generally resulted in increased editing efficiencies in eight out of nine multiplex combinations, i.e., showing a higher total editing efficiency than the combined theoretical editing of the individual RNP efficiencies, here designating a positive synergism on the total editing (Figure 3B). When targeting the 5′ end of GWD1, editing resulting for neither of the combinations did not exceed the better or the two individual RNP’s editing (Figure 3A). IDAA chromatogram of a deletion mediated by the use of two RNPs is shown in Figure 3C.

**FIGURE 4:**
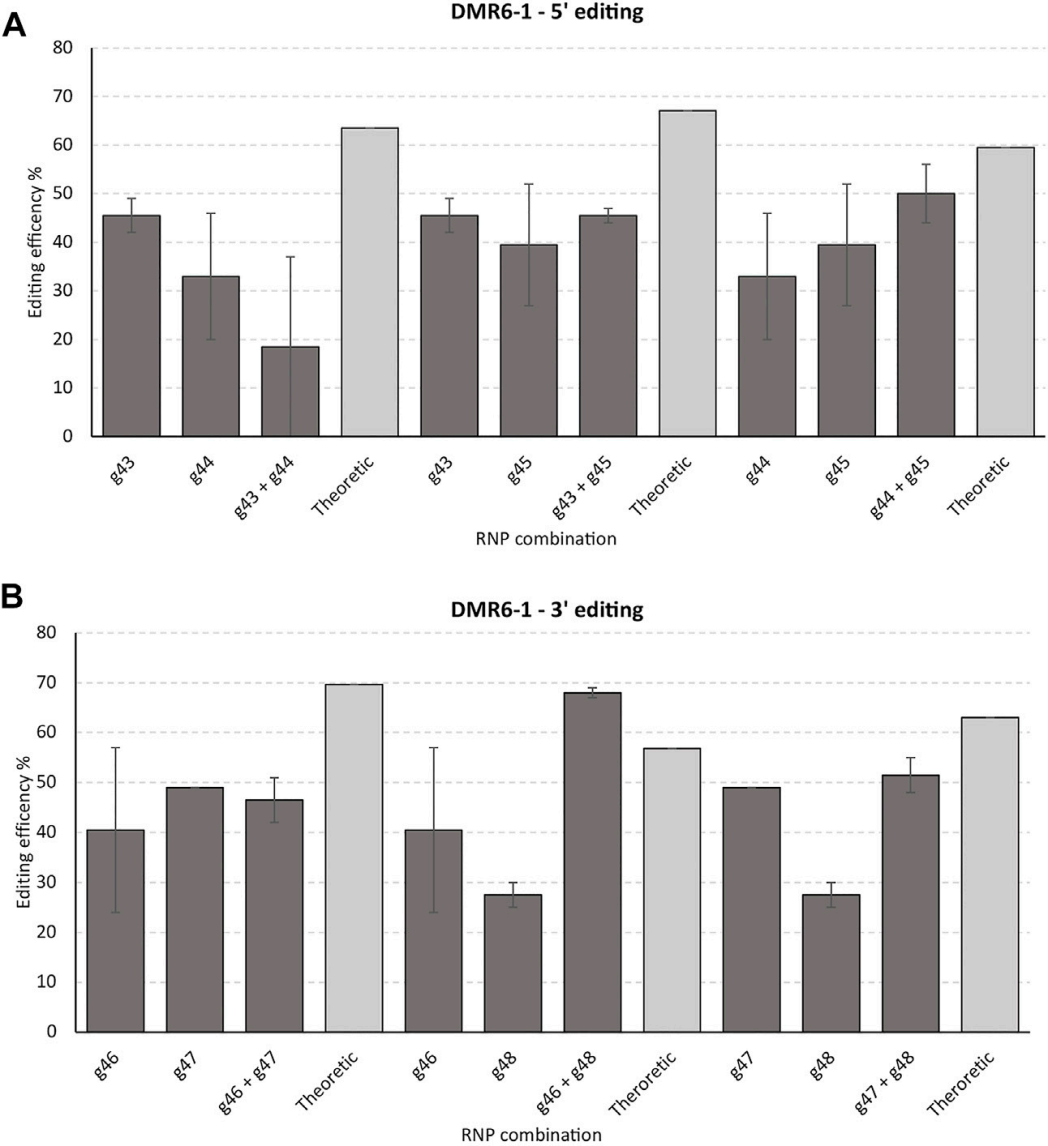
*Targeting the 5’ versus the 3’ end of DMR6-1 and effect of multiplexing*. 6 gRNAs were designed to target the 5’ (g43, g44, and g45) **(A)** and 3’ end (g46, g47, and g48) **(B)** of DMR6-1, respectively. The gRNAs were tested individually and in combination. Theoretical designates the sum of the individual two gRNAs editing’s (2 biological replicates were performed with std errors indicated). gRNAs and IDAA primers used: g43, g44, and DMR6 Forward primer 1 + DMR6 Reverse primer 1; g45 and DMR6 Forward primer 2 + DMR6 Reverse primer 2; g46, g47 & g48 and DMR6 Forward primer 3 & DMR6 Reverse primer 3 (see *Material and Methods*).

Three gRNAs targeting the 5′ end and three gRNAs targeting the 3′ end of DMR6-1 yielded from 33 to 46% and 25–49% editing of the alleles in the cell pool, respectively, as evidenced by IDAA (Figure 4A,B). Here, only one RNP combination, targeting the 3’ end of DMR6-1 (g46 and g48), resulted in a somewhat higher editing than the combined theoretical editing of the individual RNP editing efficiencies.

**FIGURE 5:**
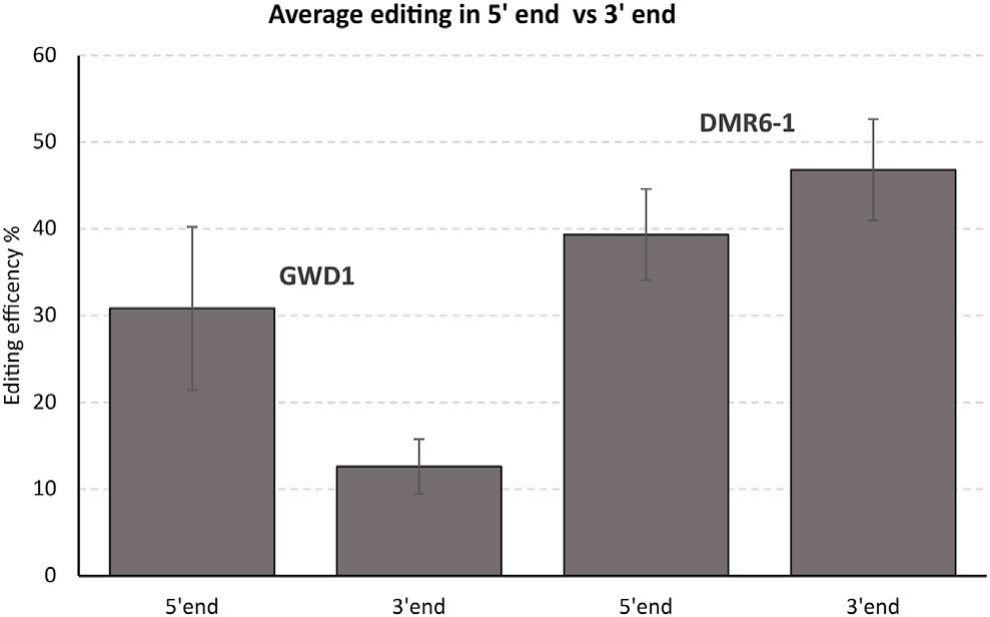
*Summarized effect of targeting the 5’ versus 3’ end of the GWD1 and DMR6-1 gene*. The average editing efficiency of the individual gRNAs targeting 5’ end and 3’ end of GWD1 and DMR6-1 (from 2 to 8 biological replicates were performed with std errors indicated).

On average, RNPs targeting the 5′ end of the GWD1 gene showed significantly higher editing (2.4 times higher, Student’s t-test, *p*-value = 0.0008) as compared to targeting the 3′ end of the gene (Figure 5). Such effect was not observed for the DMR6-1 gene (Figure 5).

### Effect of Simultaneously Targeting DMR6-1 and GWD1 Residing on Different Chromosomes

To investigate whether potential synergism in editing could also result from simultaneously targeting genes residing on different chromosomes, we monitored editing of the gRNAs gC and gD targeting the 3′ end of GWD1 (chromosome 5) and gRNA g46 and g47 targeting 3’ end of DMR6-1 (chromosome 3) transformed both individually and in combination.

**FIGURE 6:**
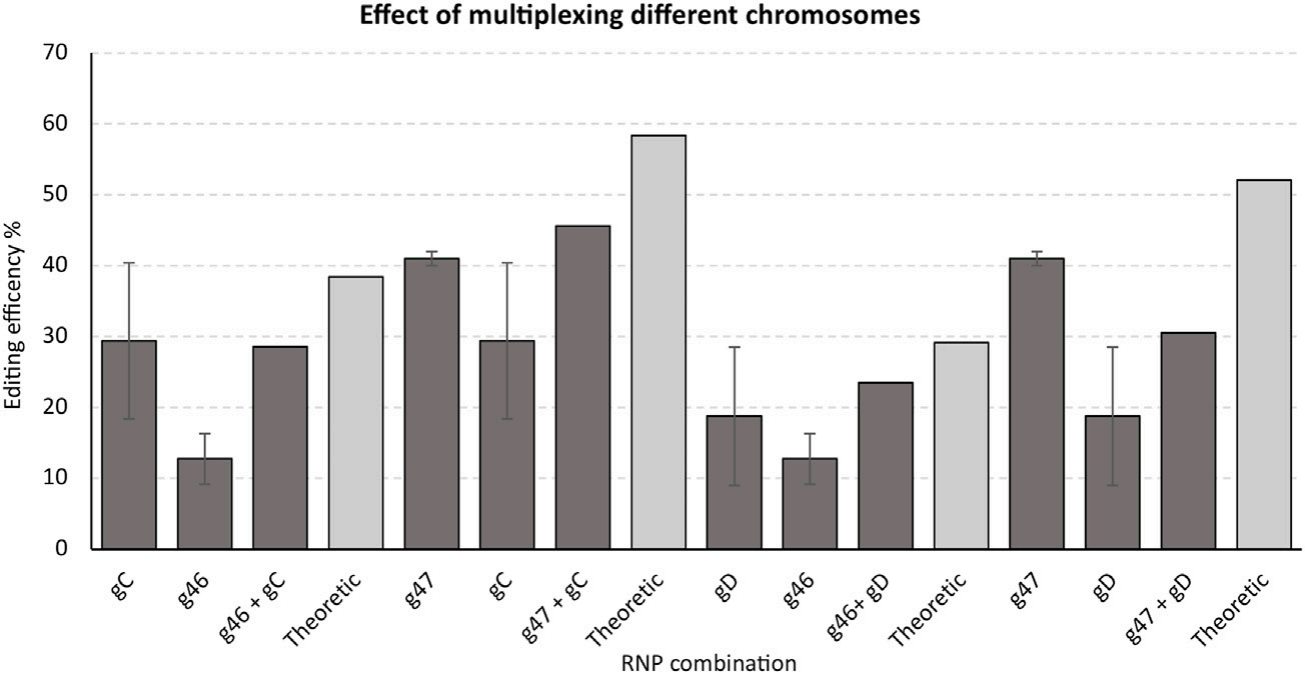
*Effect of editing when simultaneously targeting (multiplexing) the 3’ end of GWD1 and DMR6-1, residing on different chromosomes*. Single RNPs/gRNAs targeting the 3’ end of GWD1 (chromosome 5) and DMR6-1 (chromosome 3) were selected and employed individually or in combination. IDAA primer pairs used: GWD Forward primer 1, GWD Reverse primer 1 + DMR6 Forward primer 3 + DMR6 Reverse primer 3 (see *Material and Methods*).

No or a somewhat general moderate negative synergistic effect of simultaneously targeting the 3’ end of the two genes residing on different chromosomes was found (Figure 6).

### gRNA Confer Specific Indel Pattern

**FIGURE 7:**
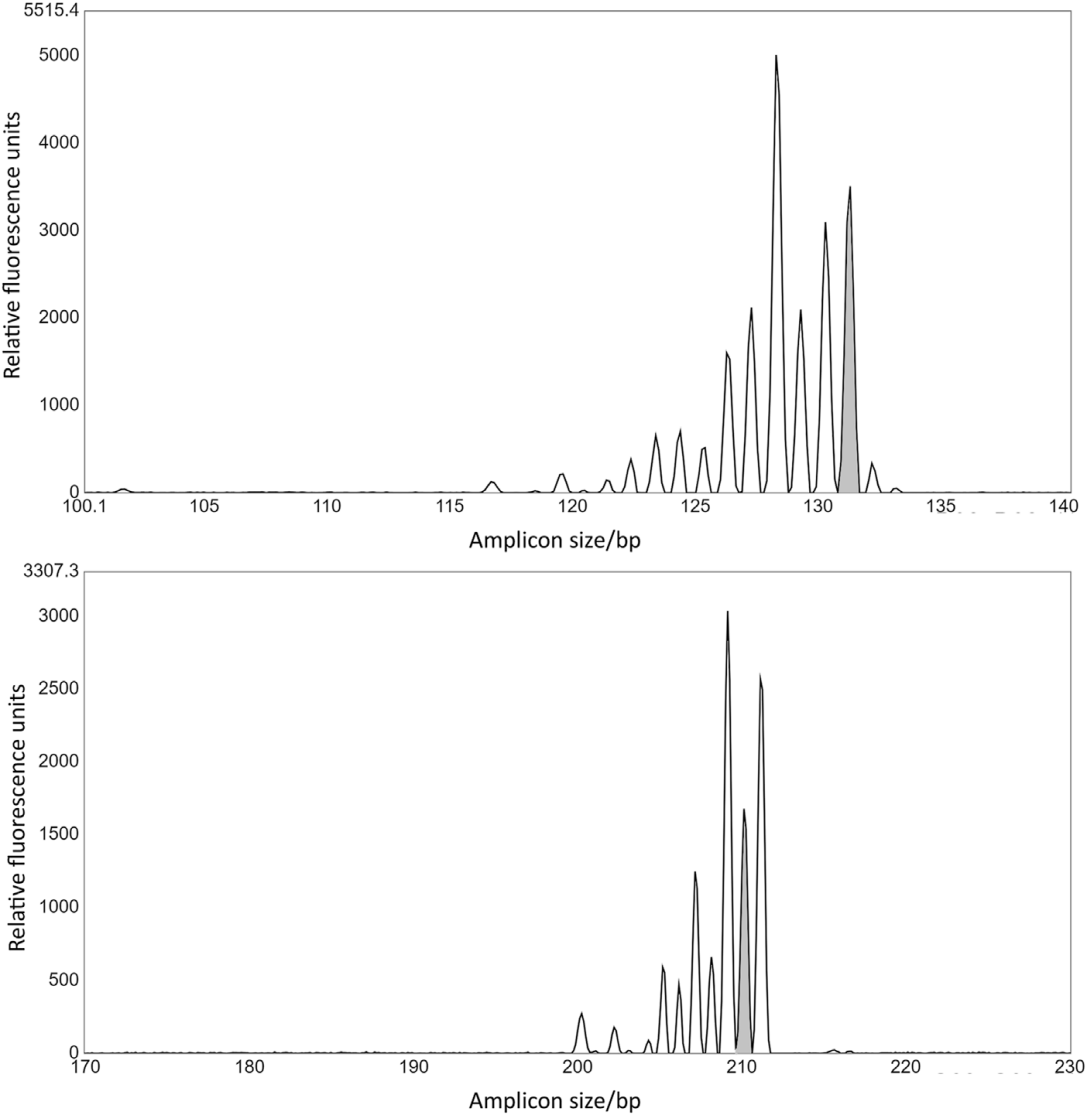
*Two DMR6-1 gRNAs confer specific indel patterns*. Two different gRNAs targeted at exon 1 in DMR6-1 were transformed into potato protoplasts and editing scored using IDAA. Grey peaks mark the wild-type amplicon. g43 confers a prevalence for a 3 bp deletion (upper panel), which in exons do not lead to frameshift of the coding sequence. g45 confers a high incidence of 1 bp indels (lower panel). These patterns were evident in repeated experiments (only data from one experiment shown). gRNA and IDAA primer pairs used: g43 and DMR6 forward primer + DMR6 reverse primer 2; g45 and DMR6 Forward primer 2 + DMR6 Forward primer 4 (see *Material and Methods*).

In mammalian cells, differences in indel patterns are normally considered a result of micro-homologous sequence around the cutting site (Grajcarek et al., 2019; Sansbury et al., 2019). Here, in agreement with this, different RNPs regularly appeared to confer individual indel patterns, as observed for the two DMR6-1 RNPs derived indel patterns shown in Figure 7.

### Comparison of gRNA in Silico Prediction Score Versus *in vivo* Editing

Following editing, *in silico* rankings and obtained *in vivo* editing efficiencies of the individual RNPs were compared to assess the most efficient design tool for potato gRNAs. While neither correlated significantly with the obtained results (Spearman rank correlation, data not shown), the SSC server correlated best, having seven out of 16 rankings fitting the *in vivo* result (Table 2).

**TABLE 2:** *In silico scoring and in vivo editing of individual gRNAs.* Efficiencies grated from highest to lowest, with one being the best scoring gRNA. Scores based on the gRNA individual score in the *in silico* prediction servers CHOPCHOP (http://chopchop.cbu.uib.no/), CRISPRater (https://crispr.cos.uni-heidelberg.de/), SSC(http://crispr.dfci.harvard.edu/SSC/). The *in vivo* score is based on editing efficiencies of single RNP/gRNA transformations as evidenced by IDAA (from two to eight biological replicates were performed with std errors indicated (see also Figures 4, 5).

	gRNA	CHOPCHOP	SSC	CRISPRater	*In vivo*
GWD 5′	gJ: TCA​GTG​GTA​AGT​ACA​GCA​TG	1	1	1	1
	gK: AGG​GAA​TAA​CTT​GCT​GTA​CC	3	4	3	4
	gL: GTT​TCG​AGG​TAA​CAG​GTT​AA	4	3	2	2
	gM: GTA​CAG​CAA​GTT​ATT​CCC​TA	2	2	2	3
GWD 3′	gA: GGA​GAG​GAG​GAA​ATT​CCT​GA	2	5	1	5
	gB: TGT​TCG​AGC​TAG​AAA​TGG​GA	3	4	5	6
	gC: GCT​GAC​CTC​CAA​GCA​AAG​GA	5	2	4	1
	gD: ATT​GGC​TGA​CCT​CCA​AGC​AA	4	3	2	2
	gE: TTT​CTG​TTC​GAG​CTA​GAA​AT	6	6	3	4
	sgI: CAC​AAC​GAC​AAC​ATA​TCC​AA	1	1	6	3
DMR6 5′	g43: TTT​GAG​GGA​GAG​TAG​AGT​GG	2	1	1	1
	g44: GTG​GCC​TAT​CGG​ATT​CGG​GT	1	2	2	3
	g45: TGG​AGA​AAT​ATG​CTC​CTG​AA	3	3	3	2
DMR6 3′	g46: GAA​GCC​ATA​GCA​GAG​AGC​CT	2	2	1	2
	g47: GAA​TTT​GGA​TCA​GTA​TGG​GC	3	1	2	1
	g48: ATC​ACC​AAG​ATT​AAT​GAC​AA	1	3	3	3

## Discussion

In agreement with our bioengineering of amylose-free starch in the cultivars Wotan and Desiree, which showed a high SNP and indel prevalence in the target Granular Bound Starch Synthase (GBSS) gene (Johansen et al., 2019), we found a 2.8 and 1.3 fold increased SNPs prevalence in the target exons of the GWD1 and DMR6-1 genes as compared to heterozygous diploid *S. tuberosum* group Tuberosum RH89-039-16 (The Potato Genome Sequencing Consortium et al., 2011) underscoring the high heterozygosity of elite potato cultivars. The high SNP and indels prevalence impose significant constraints on the placement of gRNAs and diagnostic PCR/IDAA primers. In our engineering of amylose free starch, we used non-integrative plasmid-derived transient expression of the CRISPR/Cas components delivered to the protoplasts by polyethylene glycol (PEG) transformation (Johansen et al., 2019) and experienced a high prevalence of plasmid-derived DNA fragments inserted at target cut site (Johansen et al., 2019) cooperating an earlier study where plasmid DNA was inserted during the CRISPR/Cas editing process (Andersson et al., 2018). Apart from circumventing incorporation of plasmid-derived DNA, CRISPR/Cas delivered as ribonucleoprotein (RNP) has been shown to lower off targeting events in mammalian cells (Zuris et al., 2015) as RNP is more quickly degraded, thus decreasing the window, during which the genome is exposed to CRISPR/Cas, again resulting in lower off-targeting rates (Zuris et al., 2015). These factors have prompted us and others, e.g. (Gonzalez et al., 2019), to replace plasmid delivery of the CRISPR/Cas components with RNPs.

Here we targeted the GWD1 and the DMR6-1 genes residing on chromosome 5 and 3, respectively, and estimated editing efficiencies of several gRNAs targeting the start and the latter third of the genes, here designated the 5′ and 3′ ends, as well as combining twin RNP/gRNAs (multiplexing) in the four regions. We only found a higher editing efficiency of the 5′ end of GWD1 (33 exons over a 15,414 bp region) as compared to its 3’ end. Such effect was not found for the smaller and less complex DMR6-1 (4 exons over a 6,398 bp region). Hypothetically, the DMR6-1 chromatin structure could generally be in a rather open state or may respond differently in relation to changes in the chromatin structure to the enzymatical removal of the cell wall during protoplast isolation and cell wall reconstruction in comparison to the GWD1 gene (Figure 5). In addition, DMR6-1 is compared to GWD1 encoded by a significantly smaller chromosomal region (Figures 1, 2).

We found a synergistic effect of applying twin RNPs in the 3′ end of GWD1, perhaps suggesting that generation of a Double-Stranded Break (DSB) in this region from one RNP increased the efficiency of the other. Perhaps, as seen when combining restriction enzymes for digesting super-coiled plasmid DNA, where initial digestion by one enzyme with concomitant relaxation of the pDNA may aid the digestion activity of the other, typically a less efficient cutter. Such an effect was not found in multiplexed targeting of both regions in DMR6-1 and the 5’ end of GWD1. We do not have plausible explanations for this differential behavior. It should be noted that the contribution of CRISPR/Cas re-cut of a perfectly repaired DSB is blocked when a deletion is made, and the extent of this is difficult to evaluate.

To investigate whether a RNP targeted at one chromosome might influence the efficiency of a RNP targeted at another chromosome, we selected the most efficient RNP/gRNA targeting the 3′ end of GWD1 (chromosome 5) and the most efficient RNP/gRNA targeting the 3′ end of DMR6-1 (chromosome 3), and performed transformations targeting the genes individually or simultaneously. Simultaneous targeting of the 3′ end of both genes yielded no or a slightly negative effect on the individual RNP/gRNA editing efficiencies when compared to the editing efficiencies of the single RNP/gRNA transformations (Figure 6). Underlying mechanisms for these observations remain highly speculative.

Unraveling potential synergistic effects on editing, positive as negative, are of great practical importance for successful and manageable multigene precision breeding in plants, including crops. This study embodies a first very limited attempt to assess gene editing efficiencies in relation to target gene structure and hypothetical chromatin status, here confined to gene editing of two unrelated genes with single and combined RNPs/gRNAs. While much larger experimental set-ups, including a significantly higher number of target genes having a variety of functions within the cell/organism, are needed to reveal the nature of such relations, this study may provide a framework for developing such larger scale experimental strategies.

## Data Availability

The original contributions presented in the study are included in the article, further inquiries can be directed to the corresponding author. Cultivar and allele specific sequence data for the target regions in GWD1 (Saturna) and in DMR6-1 (Wotan) are included in Figure 1 and 2, respectively.
