# Corrigendum: Strategies for Efficient Gene Editing in Protoplasts of *Solanum tuberosum* Theme: Determining gRNA Efficiency Design by Utilizing Protoplast (Research)

**DOI:** 10.3389/fgeed.2022.914100

**Published:** 2022-08-04

**Authors:** Frida Meijer Carlsen, Ida Elisabeth Johansen, Zhang Yang, Ying Liu, Ida Nøhr Westberg, Nam Phuong Kieu, Bodil Jørgensen, Marit Lenman, Erik Andreasson, Kåre Lehmann Nielsen, Andreas Blennow, Bent Larsen Petersen

**Affiliations:** ^1^ Department of Plant and Environmental Sciences, Faculty of Science, The University of Copenhagen, Copenhagen, Denmark; ^2^ Kartoffel Mel Centralen Amba, Brande, Denmark; ^3^ Department of Cellular and Molecular Medicine, Faculty of Health Sciences, University of Copenhagen, Copenhagen, Denmark; ^4^ Department of Plant Breeding, Swedish University of Agricultural Sciences, Alnarp, Sweden; ^5^ Department of Plant Protection Biology, Swedish University of Agricultural Sciences, Alnarp, Sweden; ^6^ Bioscience, Aalborg University, Aalborg, Denmark

**Keywords:** ribonucleoprotein/gRNA design, gene editing (CRISPR/Cas9), protoplast, gRNA efficiency, multiplexing, complex genome, potato

In the original article, there were various errors present throughout the main text. These errors have been corrected in the original article.

Additionally, Figure 1 and Table 1 have been updated. The updated figure and table are shown below. The Funding statement has also been updated and is shown below.

**FIGURE 1:**
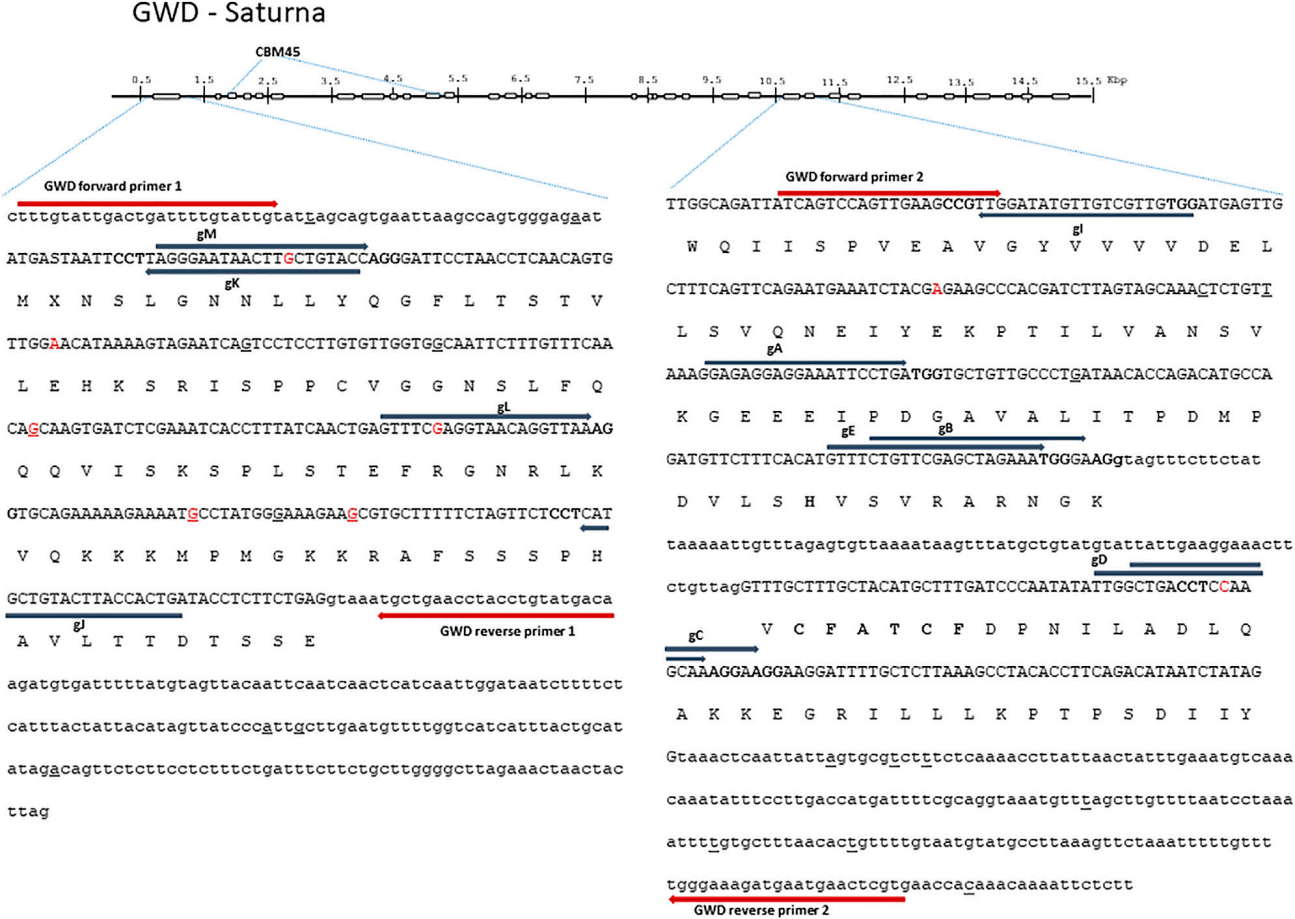
Glucan Water Dikinase (GWD) 1—structure and full allelic sequence of gRNA target regions. Overall gene structure with exons (boxes) and the area containing carbohydrate-binding module (CBM) depicted above. Left: the nucleotide sequence of exon 1 and introns. Right: exon 24 and 25 including introns. Exons are depicted in capital letters with the amino acid sequence indicated. Small nucleotide polymorphisms (SNPs) from cultivars included in the SPUD database are marked with red, and SNPs found in Saturna are underlined. Grey arrows designate gRNAs (gA, gB, gC, gD, gE, gI, gJ, gK, gL, and gM) with PAM sites marked in bold. Red arrows designate diagnostic IDAA PCR primers. The “CFATC” region, containing cysteine’s hypothesized to be involved in inter or intra-di-sulfide bond formation and thus in putative redox-state modulation of GWD activity is marked with bold. The active site histidine residue is also marked with bold.

**TABLE 1:** gRNAs and diagnostic IDAA primers for each of the four target regions. Scores and first selection of gRNAs were obtained by feeding ca 1 kb regions to the *in silico* prediction servers CHOPCHOP (http://chopchop.cbu.uib.no/), CRISPRater (https://crispr.cos.uniheidelberg.de/) and SSC (http://crispr.dfci.harvard.edu/SSC/).

	Diagnostic PCR(s)	gRNA
GWD—5′ exon 1	GWD Forward primer 1	gJ: TCA​GTG​GTA​AGT​ACA​GCA​TG
	5′ TTT​GTA​TTG​ACT​GAT​TTT​GTA​TTG​T 3′	gK: AGG​GAA​TAA​CTT​GCT​GTA​CC
	GWD Reverse primer 1	gL: GTT​TCG​AGG​TAA​CAG​GTT​AA
	FAM 5′ TAG​TTT​CTA​AGC​CCC​AAG​CA3′	gM: GTA​CAG​CAA​GTT​ATT​CCC​TA
GWD—3′ exon 24 + 25	GWD Forward primer 2:	gA: GGA​GAG​GAG​GAA​ATT​CCT​GA
	5′ TCA​GTC​CAG​TTG​AAG​CCG​TTG 3′	gB: TGT​TCG​AGC​TAG​AAA​TGG​GA
	GWD Reverse primer 2:	gC: GCT​GAC​CTC​CAA​GCA​AAG​GA
	FAM 5′ TCA​CGA​GTT​CAT​TCA​TCT​TTC​CCA 3′	gD: ATT​GGC​TGA​CCT​CCA​AGC​AA
		gE: TTT​CTG​TTC​GAG​CTA​GAA​AT
		gI: CAC​AAC​GAC​AAC​ATA​TCC​AA
DMR6—5′ exon 1	DMR6 Forward primer 1	g43: TTT​GAG​GGA​GAG​TAG​AGT​GG
	FAM 5′ CCA​TGG​AAA​CGA​AAG​TTA​TTT​C 3′	g44: GTG​GCC​TAT​CGG​ATT​CGG​GT
	DMR6 Reverse primer primer 1	
	5′ CAA​CCT​AAG​TCA​ATT​ATT​GGA​AC 3′	
DMR6—5′ exon 2	DMR6 Forward primer 2	g45: TGG​AGA​AAT​ATG​CTC​CTG​AA
	*5′ AGC​TGA​CCG​GCA​GCA​AAA​TTG*GGT​AGC​TGG​GGA​ATT​TTT​CA 3′	
	DMR6 Reverse primer 2	
	5′ GGT​TAC​CAT​GCA​TAA​CTA​TAC​ACA​C 3′	
	FAM primer	
	FAM 5′ AGC​TGA​CCG​GCA​GCA​AAA​TTG 3′	
DMR6—5′ exon 1 + 2	DMR6 Forward primer 1	g43: TTT​GAG​GGA​GAG​TAG​AGT​GG
	FAM 5′ CCA​TGG​AAA​CGA​AAG​TTA​TTT​C′3	g44: GTG​GCC​TAT​CGG​ATT​CGG​GT
	DMR6 Reverse primer 2	g45: TGG​AGA​AAT​ATG​CTC​CTG​AA
	5′ GGT​TAC​CAT​GCA​TAA​CTA​TAC​ACA​C 3′	
	DMR6 Reverse primer 4	
	FAM 5′ CGA​TGG​ATT​AGA​AGG​CCA​TTC 3′	
DMR6—3′ exon 3	DMR6 Forward primer primer 3	g46: GAA​GCC​ATA​GCA​GAG​AGC​CT
	5′ ATC​GTG​AGC​AGA​TAT​TGC​ACG 3′	g47: GAA​TTT​GGA​TCA​GTA​TGG​GC
	DMR6 Reverse primer 3	g48: ATC​ACC​AAG​ATT​AAT​GAC​AA
	FAM 5′ GGT​TTA​CCT​GCA​ATT​GAT​CAC 3′	

